# Thermal versus photochemical tautomerization of cytosine and guanine: a BLYP computational study along the IRC curves

**DOI:** 10.55730/1300-0527.3490

**Published:** 2022-08-04

**Authors:** Tsvetina D. CHERNEVA, Vassil B. DELCHEV

**Affiliations:** Department of Physical Chemistry, University of Plovdiv, Plovdiv, Bulgaria

**Keywords:** DFT calculations, excited states, nucleic acid bases, tautomerizations

## Abstract

We performed a theoretical study, at the BLYP/aug-cc-pVDZ theoretical level, of the mechanisms of H-transfer between biologically relevant tautomers of cytosine and guanine. The analysis of the ground- and excited state energy barriers of the conversions showed that in some cases they are lower as compared to the ground state reactions. The inclusion of one water molecule as a catalyst in the oxo-amino form of cytosine and guanine drastically changes the mechanism of the reactions in the ground as well as in the excited state. For example, the ground state reaction path of cytosine reduces the energy barrier from 165 to 60 kJ.mol^−1^ and for guanine, this reduction is from 92 kJ.mol^−1^ to 24 kJ.mol^−1^. The results obtained are in support to the high photostability of the nucleic acid bases which guarantee the normal biological function of nucleic acids.

## 1. Introduction

Cytosine and guanine are the main building blocks in the macromolecules of nucleic acids. They play a crucial role in the processes of encoding and translation of genetic information in living world. These two bases participate in the so-called Watson-Crick (WC) model in which they are bound through H-bonds and in this way they are present in the macromolecule of DNA [1]. Usually in the WC model cytosine and guanine participate with their amino oxo tautomers [2]. For cytosine one evidence in this aspect is the registered C=O stretching vibration in the IR spectrum of compound within the WC base pair [3]. The generation of other tautomers could seriously disturb the normal biofunctioning of the macromolecules of RNA and DNA. Therefore, the tautomerization processes of the two nucleobases and their control is of significant importance.

Cytosine is a pyrimidine nucleobase whose absorption spectrum, at first sight, is relatively simple, comprising basically two broad and intense absorption bands. The maxima of the bands registered at different conditions are summarized in Table 1.

It has been shown that the main absorption maxima can be assigned for the π→π* transitions in the amino oxo form of cytosine [9]. This nucleobase is photostable while its close structural analogue isocytosine is not. Recently we performed a comparative computational study, at the CC2 level, of the cytosine and isocytosine and it has been found that isocytosine tautomerizes to amino-hydroxy form through the ^1^πσ* excited state [10]. A decade earlier Vranken et al. have demonstrated that UV irradiation (λ = 308 nm) of matrix-isolated isocytosine leads to the tautomerization of the amino-oxo tautomer to the amino-hydroxy form [11].

Guanine is a purrine base that shows one intense band in water solution near 250 nm (4.96 eV) and a weaker one at 275 nm (4.51 eV) [12–14]. Mishra et al. [14] have reported calculated UV bands of different tautomers of guanine. Their experimental research has revealed two strong peaks near 350 nm and a weak shoulder near 450 nm in the fluorescence spectrum of guanine water solution obtained with excitation at 250 nm (4.96 eV) and 275 nm(4.51 eV) [14]. Quantum chemical calculations of the biologically relevant keto-N9H tautomer of guanine have shown that the UV excitation leads to the population of the ^1^ππ* excited state [15,16].

Our previous DFT investigations revealed that the proton transfers in single bases are facilitated by using a water molecule as a catalyst [17,18]. The energy barriers of the ground-state proton transfers in cytosine are drastically reduced when water molecules are involved in the intermolecular proton transfers [18,19]. The same fact was established for guanine either [17]. Recently we tested the BLYP functional for studying excited state reaction mechanisms in pyrimidines and we found that the results are promising and some energy quantities are close to their experimental values, e. g., UV absorption maxima [20,21].

Regarding the accuracy of the DFT methods one can find in the scientific space a lot of investigations comparing the results obtained at the DFT level with experimental values and electron correlation level [20,22–25]. For example the DFT functionals are compared with results obtained at the ADC(2) method for pyridine-thiophene oligomers [22]; BLYP and B3LYP functionals have been tested for nucleobase stacks [24]; spectroscopic properties of cytosine have been discussed [26,27] etc. The BLYP functional has been tested to give accurate enough results for predicting the UV absorption spectra of 6-azauracil in water media [20].

Obviously, cytosine and guanine are chromophores in macromolecules of nucleic acids and the knowledge of their phototransformations is of great importance for predicting and controlling some unwished reactions provoked by the sunlight. Thus the aim of the current research is to check the possibility of tautomers of cytosine and guanine to isomerize through excited-state reaction paths along the IRC curves of tautomeric mechanisms.

## 2. Theoretical methods

The research includes only those tautomers of cytosine and guanine which can be potentially formed when nucleobases are segments of corresponding nucleosides. It means that in all studied here tautomers we left one H atom linked to N1 of cytosine and guanine ring since this position in nucleosides is engaged with a sugar residue. In other words, this H-atom will not be involved in tautomerization processes under study.

The ground-stat equilibrium geometries of the studied tautomers of cytosine and guanine were optimized in the gas phase at BLYP level of theory using the aug-cc-pVDZ basis set. Further frequency calculations were carried out to prove that the optimized structures are true minima. No imaginary frequencies were found. The tautomerization reactions of the isomers of the two compounds were studied at the same theoretical level. The thermal transition states of the ground-state reactions were optimized as first order saddle points–with one imaginary frequency each. The form of the imaginary frequency corresponds to a motion of the proper H-atom in order to form one of the two tautomers involved in a given mechanism.

Ground-state IRC calculations were performed with each transition state in both directions: towards the product and towards reactant. The generated geometries along each thermal reaction path were used for subsequent calculations of the excited states, at the TD BLYP level, lying vertically over each structure. The vertical excitation energies of the tautomers minima were also calculated using the ground-state equilibrium geometries of the tautomers.

All calculations were performed at the (TD)BLYP/aug-cc-pVDZ theoretical level using the Gaussin 03 program package [28]. The selected here DFT functional has been tested for an enormous number of organic compounds [29–31]. It has been demonstrated that this functional predicts excitation energies that are close to the experimental absorption maxima of many organic compounds [21]. The geometries of the studied structures were visualized with the Chemcraft software [32].

## 3. Results and discussion

**Figure 1:**
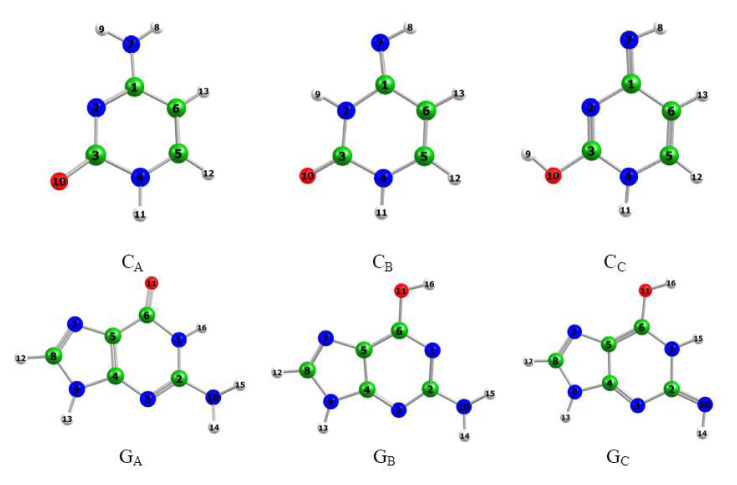
Optimized ground-state equilibrium geometries of cytosine and isocytosine included in the research.

The gas phase ground-state equilibrium geometries of the cytosine and guanine involved in the study are illustrated in Figure 1.

The ground-state equilibrium geometries of amino tautomers of cytosine and guanine exhibit pyramidal behavior of the amino group. This can be seen from the sum of the bond angles (∑) around N7: ∑ = 358.6° for C_A_, ∑ = 345.1° for G_A_, and ∑ = 352.2° for G_B_. Our attempts to optimize planar structures of these tautomers failed since they are not true minima: they show imaginary vibrational modes connected with out-of-plane vibrations of the amino group H-atoms.

The calculated vertical excitation energies of the tautomers are listed in Table 2. The data show that the lowest excited state for all tautomers, except C_A_, is the spectroscopically active ^1^ππ^*^ one (4.04 eV), which could be directly populated through an optical transition (excitation) from the ground state of a concrete tautomer. This electronic state is extremely low in energy for tautomer G_C_ which implies high photochemical activity of the compound. Only for tautomer C_A_ the lowest-lying excited state is the ^1^nπ^*^ excited state (3.69 eV). As known the dark ^1^ps^*^ excited state is the driven state for mechanisms connected with H-transfers–like tautomerizations [33,34]. The lowest-lying ^1^ps^*^ excited state (3.14 eV) was calculated for tautomer G_C_.

**Figure 2:**
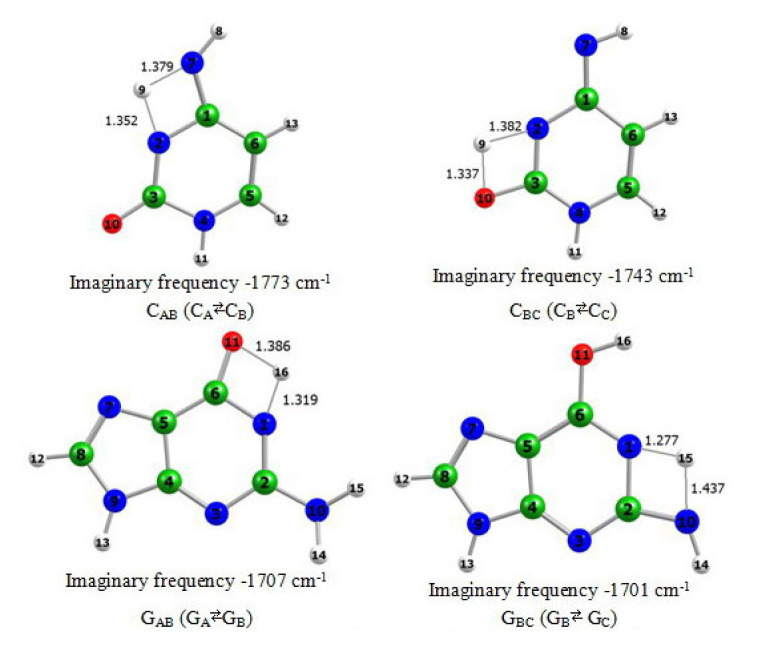
Optimized transition states of the tautomerizations of cytosine and guanine at the BLYP/aug-cc-pVDZ level of theory.

In order to study the proton transfer reactions we optimized the thermal transition states of the tautomeric conversions. Their structures are presented in Figure 2.

The analysis of each imaginary frequency showed that the found saddle point is a real transition state of the corresponding tautomerization. The form of each imaginary frequency describes the motion of the proper H-atom towards the formation of one of the tautomers involved in the process. The energy barriers of the conversions are listed in Table 3.

**Figure 3:**
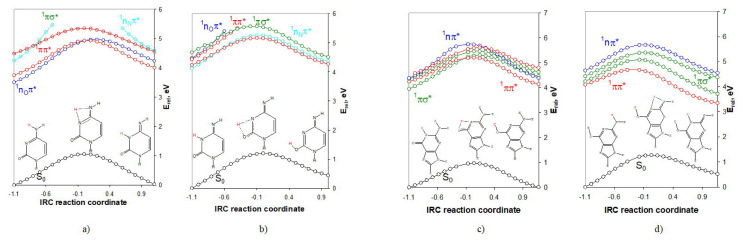
IRC excited-state reaction curves of the tautomeric conversions: a) C_A_⇄C_B_; b) C_B_⇄C_C_; c) G_A_⇄G_B_; d) G_B_⇄G_C_. The relative energies are referenced to the energy of the ground-state equilibrium geometry of tautomer: a) C_A_ (−394.860671 a.u.); b) C_B_ (−394.850855 a.u.); c) G_A_ (−542.479633 a.u.); and d) G_B_ (−542.462721 a.u.); all found at the BLYP/aug-cc-pVDZ theoretical level

The data show considerably high energy barriers in the gas phase, and one can suppose that the reactions would occur somehow easier in excited state. To check this assumption, we performed IRC calculations, starting from each transition state. With the generated geometries along each reaction path we calculated the vertical excitation energies in order to construct the excited-state reaction paths. The results are summarized in Figure 3.

Since the expected excited-state reaction paths responsible for the interconversions are those of the spectroscopically active states (^1^ππ^*^, ^1^nσ^*^) below we shall comment these curves only. Moreover, as it is known the H-detachment/attachment processes occurring by the so-called PIDA (phot-induced dissociation-association) mechanism [28,29] are expected to proceed along the driven ^1^πσ^*^ excited state. That is why we will also include the ^1^πσ^*^ excited-state reaction paths in the discussion.

Figure 3a shows that the transformation C_A_→C_B_ should occur along the reaction curves of the two ^1^ππ^*^ excited states. However, the reaction along the curve of the lowest-lying ^1^ππ^*^ excited state shows a rise of the energy barrier with 0.12 eV. The proceeding of the photoreaction in the second excited state shows a reduction of the energy barrier with 0.23 eV, both compared to the thermal reaction (in the ground state).

The subsequent transformation of tautomer C_B_ into C_C_ (Figure 3b) can happen through the ^1^ππ^*^ and ^1^πσ^*^ excited-state reaction paths. In both cases the energy barriers of the photoreactions are reduced with 0.28 eV and 0.33 eV as compared to the ground state process. Thus, from a kinetics point of view, the photoreaction is favored as compared to the ground state one.

For the transformation of G_A_→G_B_ (Figure 3c) it is observed a reduction of the energy barrier with 0.01 eV along the first ^1^ππ^*^ excited state, whereas along the second ^1^πσ^*^ excited state the energy barrier is increased with 0.37 eV as compared to the ground state. As for the transformation G_B_→G_C_ (Figure 3d) we observed a reduction of the energy barriers along the ^1^ππ^*^ (with 0.68 eV) and ^1^πσ^*^ (with 0.41 eV) excited-state reaction paths that facilitate the H-transfers as compared to the thermal reaction.

**Figure 4:**
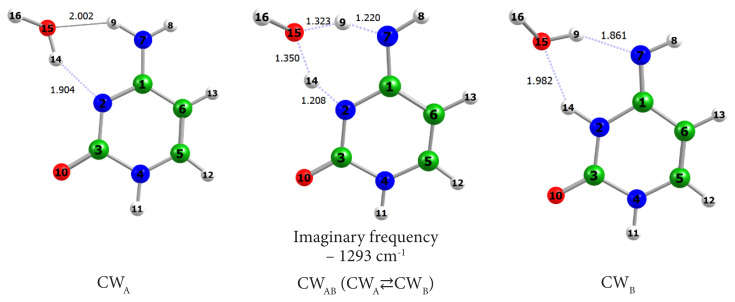
Ground-state equilibrium geometries of the water complexes of cytosine CW_A_ and CW_B_ and the transition state between them −BLYP/aug-cc-pVDZ level.

In order to check how one water molecule (as a catalyst) could change the tautomeric mechanisms we optimized the ground-state equilibrium geometries of the water complexes of tautomers C_A_ and C_B_ and the transition state standing on the path of their mutual interconversion. The structures are illustrated in Figure 4. In fact, for cytosine and guanine we included in the water assisted proton transfers only these tautomers that can be obtained directly from the most stable and abundant tautomers in the DAN/RNA macromolecules.

**Figure 5:**
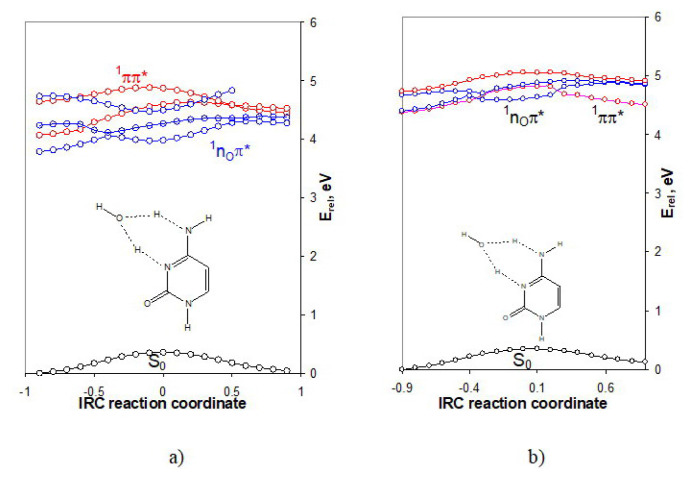
IRC excited-state reaction curves of the tautomeric conversions CW_A_⇄CW_B_ a) in the gas phase, and b) in water surroundings (PCM). The relative energy is referenced to the energy of the ground-state equilibrium geometry of tautomer WC_A_ (−471.317065 a.u. and −471.336044 a.u.).

The performed IRC calculations with the transition state and a subsequent calculation of the excited states along the IRC showed that the water molecule-catalyst drastically changes the energy barrier of the thermal reaction as well as the mechanism of the photoreaction (Figure 5a). With regards to the thermal reaction we found a reduction of the energy barriers of the forward reaction to about 64% (from 165 to 60 kJ.mol^−1^) and of the reverse reaction to about 68% (from 158 to 50 kJ.mol^−1^).

The driven state of the photochemical reaction in this case is the dark ^1^nπ^*^ excited state, instead of the typical for such cases ^1^πσ^*^ one. The dark ^1^nπ^*^ excited state can be populated by the low-lying spectroscopically active ^1^ππ^*^ excited state.

The inclusion of the solvent model (PCM, Figure 5b) affects only the energy barrier of the ^1^ππ^*^ excited state. It is reduced with 12 kJ.mol^−1^ as compared to the same in the gas phase. The ^1^nπ^*^ excited-state reaction paths stay almost unchanged. Moreover, in the beginning of the reaction coordinate the ^1^ππ^*^ excited state is almost degenerated with the ^1^nπ^*^ excited state. The water-supported H-transfers in other tautomers and nucleobases are objects of our future research.

**Figure 6:**
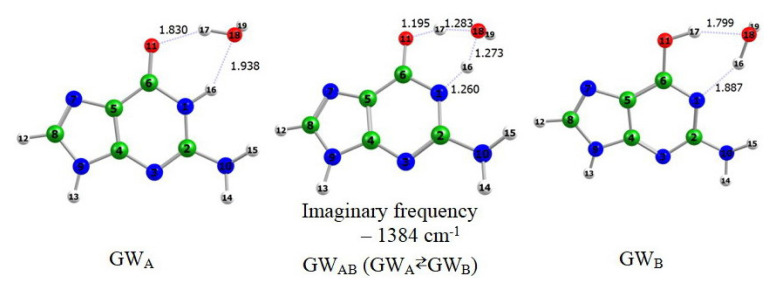
Ground-state equilibrium geometries of the water complexes of guanine.

In Figure 6 are given the optimized structures of the water complexes of tautomer A and B of guanine and the transition state standing between them. The transition state GW_AB_ of the reaction was found as a first-order saddle point with one imaginary frequency whose form describes the tautomerization process.

**Figure 7:**
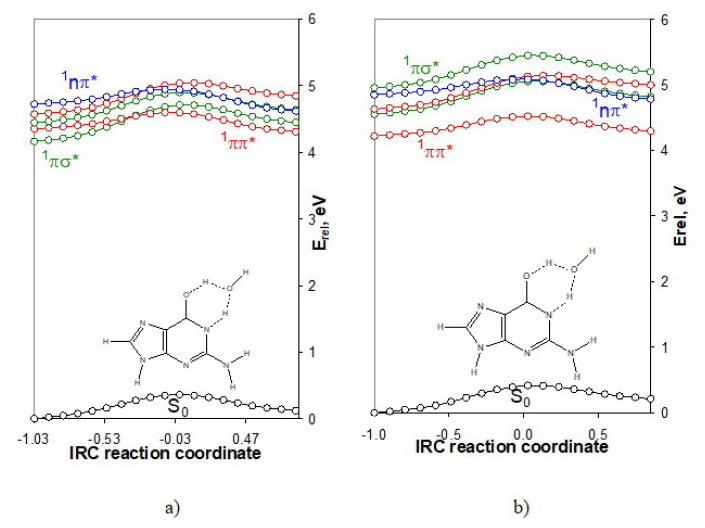
IRC excited-state reaction curves of the tautomeric conversions GW_A_⇄GW_B_ a) in the gas phase, and b) water surroundings (PCM). The relative energy is referenced to the energy of the ground-state equilibrium geometry of tautomer GW_A_ (−618.933331 a.u. and −618.954878 a.u.).

The water-assisted proton transfer in guanine in the gas phase and water surroundings are described in Figure 7. The gas phase mechanism shows that two excited states have competed for the driven state–the bright ^1^ππ^*^ excited state and the repulsive ^1^πσ^*^ one. In a water environment, the driven state is only the ^1^ππ^*^ one.

The reduction of the energy barriers along the ^1^ππ^*^ excited-state reaction path as compared to the intramolecular proton transfer is 74% (from 92 kJ.mol^−1^ to 24 kJ.mol^−1^) for the gas phase mechanism and 68% for the mechanism modeled by PCM. The energy barrier of the ground state is reduced by 64% (from 94 to 34 kJ.mol^−1^) which is exactly the same as for cytosine.

## 4. Conclusion

The computational study (BLYP/aug-cc-pVDZ) of the proton transfers in the biologically relevant tautomers of cytosine and guanine led to the next major conclusions: in some cases, the photoreactions along the ^1^ππ^*^ and ^1^πσ^*^ excited-state reaction paths are higher as compared to the ground state. The presence of energy barriers along the excited state curves are in support of the high photostability of the nucleic acid bases, a fact which is important for the normal biological function of nucleic acids. A drastic reduction of the energy barriers of the photoreactions can be achieved when a water molecule mediates the H-transfer in the cytosine oxo-amino forms of cytosine and guanine. The driven state of the photochemical reaction of cytosine in this case is the dark ^1^nπ^*^ one. The water-assisted proton transfer in guanine occurs along the ^1^ππ^*^ excited-sate reaction path. The studied mechanisms showed a lot of crossing points between the energy curves of the excited states that could serve as “switch” channels for the population of these states and subsequent run of the reactions along one preferred minimum energy path.

## Figures and Tables

**Table 1:** UV absorption bands of cytosine [4].

Surroundings	maxima in eV	
Vapor [5]	4.3	
Water (pH = 7) [6,7]	4.7	5.3
Ethanol [8]	4.5	5.2

**Table 2:** Vertical excitation energies (eV) of the tautomers of cytosine and guanine in the gas phase. The oscillator strenghts of the spectroscopically active states are given in brackets.

Tautomer C_A_		Tautomer C_B_		Tautomer C_C_	
^1^np^*^	3.69	^1^pp^*^	4.28 (0.0597)	^1^pp^*^	3.95 (0.0013)
^1^pp^*^	4.04 (0.0193)	^1^np^*^	4.38	^1^np^*^	4.03
^1^np^*^	4.39	^1^ps^*^	4.61	^1^ps^*^	4.11
^1^ps^*^	4.53	^1^np^*^	4.63	^1^ns^*^	4.53 (0.0018)
^1^ns^*^	4.63 (0.0017)	^1^pp^*^	4.68	^1^ps^*^	4.63
^1^pp^*^	4.76 (0.0587)	^1^ps^*^	5.28	^1^np^*^	4.73
^1^pp^*^	4.84 (0.0032)	^1^ns^*^	5.29 (0.0102)	^1^ps^*^	4.96
^1^np^*^	4.94	^1^ps^*^	5.36	^1^ns^*^	5.07 (0.0084)
^1^np^*^	5.02	^1^pp^*^	5.49 (0.1936)	^1^np^*^	5.07
^1^ps^*^	5.09	^1^ps^*^	5.49	^1^pp^*^	5.11 (0.1677)
Tautomer G_A_		Tautomer G_B_		Tautomer G_C_	
^1^pp^*^	3.93 (0.0119)	^1^pp^*^	4.13 (0.0913)	^1^pp^*^	2.92 (0.0298)
^1^pp^*^	4.34 (0.0764)	^1^pp^*^	4.32 (0.0007)	^1^ps^*^	3.14
^1^ps^*^	4.43	^1^np^*^	4.45	^1^ps^*^	3.92
^1^np^*^	4.52	^1^ps^*^	4.51	^1^np^*^	3.96
^1^pp^*^	4.57	^1^ps^*^	4.76	^1^np^*^	4.17
^1^np^*^	4.72	^1^pp^*^	4.82 (0.0808)	^1^pp^*^	4.34 (0.0917)
^1^np^*^	4.78	^1^np^*^	5.13	^1^ns^*^	4.58 (0.0005)
^1^np^*^	4.85	^1^np^*^	5.18	^1^ps^*^	4.60
^1^ps^*^	4.91	^1^np^*^	5.26	^1^ps^*^	4.77
^1^np^*^	5.05	^1^ps^*^	5.27	^1^ns^*^	4.78 (0.0128)

**Table 3:** Ground-state energy barriers of the tautomer interconversions, kJ.mol^−1^ (BLYP/aug-cc-pVDZ).

Conversion	Forward	Reverse
**Cytosine**		
C_A_⇄C_B_	165	158
C_B_⇄C_C_	197	115
**Guanine**		
G_A_⇄G_B_	140	134
G_B_⇄G_C_	208	102
